# Effects of Cryopreservation on Canine Multipotent Stromal Cells from Subcutaneous and Infrapatellar Adipose Tissue

**DOI:** 10.1007/s12015-015-9634-4

**Published:** 2015-11-04

**Authors:** Wei Duan, Mandi J. Lopez

**Affiliations:** Laboratory for Equine and Comparative Orthopedic Research, Department of Veterinary Clinical Sciences, School of Veterinary Medicine, Louisiana State University, Baton Rouge, LA USA

**Keywords:** Adipose, Infrapatellar, Cryopreservation, Subcutaneous, Canine, Multipotent stromal cells

## Abstract

Adipose derived multipotent stromal cells (ASCs) isolated from brown versus white adipose tissues, may have distinct in vitro properties, including response to cryopreservation, due to differences in tissue physiology. This study was designed to determine the ultrastructure, immunophenotype, in vitro expansion capabilities and multipotentiality of paired canine ASCs harvested from subcutaneous (SUB) and infrapatellar (IFP) adipose tissue up to cell passage (P) 3 before and after cryopreservation. Adipocyte and ASC ultrastructure from the same tissue were distinct, and morphologies of both differed between tissue sources and with cryopreservation. Cell expansion and colony forming unit frequencies were similar between ASCs from both tissue sources before and after cryopreservation. Most fresh cells were CD29+, CD44+, CD90+ and CD34− up to P3. Cryopreserved P1 and P3 cells had lower percentages of CD29+ and 44+ cells, respectively, compared to fresh. Peroxisome proliferator-activated receptor γ (PPAR-γ) gene expression and sex determining region Y-box 2 (SOX2), CD29 and CD44 protein expression was lower in cryopreserved versus fresh P3 ASCs. Both PPAR-γ and osteopontin (OPN) protein expression increased in fresh and cryopreserved P3 ASCs cultured in adipogenic and osteogenic induction medium, respectively, while SOX2 decreased. Based on the study findings, in vitro expansion and multipotentiality are not distinct among canine SUB and IFP ASCs before or after cryopreservation. However, cryopreservation alters ASC ultrastructure, immunophenotype and transcription factor expression from both tissue sources. Future studies are necessary to determine the impact of cryopreservation on cell potential for therapy and de novo tissue generation.

## Introduction

Tissue engineering with adult multipotent stromal cells (MSCs) harvested from adipose tissue, adipose derived multipotent stromal cells (ASCs), is rapidly emerging as a therapeutic reality in human and veterinary medicine [1, 2]. Higher MSC tissue density and comparable cell plasticity to bone marrow derived multipotent stromal cells are some of the characteristics that establish adipose tissue as a viable MSC source [3]. Existing knowledge supports potential differences in ASC behavior among adipose depots in numerous species [4, 5]. One rationale for observed differences is varying proportions of brown and white adipose tissue among harvest sites [6]. Intra-articular and visceral adipose tissue are predominantly white while subcutaneous adipose tissues are mostly brown [7]. Distinctions between cells isolated from brown versus white adipose tissue like lower plasticity of brown adipose tissue cells, have been attributed to epigenetic factors and functional differences, endocrine versus thermogenic of white and brown, respectively [8, 9]. Distinct features of cells isolated from brown and white adipose tissues contribute to efforts to optimize isolation procedures and culture conditions for cells from each [10].

Canine infrapatellar adipose tissue ASCs reportedly have similar in vitro properties as bone marrow derived multipotent stromal cells and higher expansion capacity and plasticity than undifferentiated cells from joint capsular and cranial cruciate ligament synovium [11]. This makes them appealing for intra-articular tissue regeneration. Subcutaneous adipose tissue harvest is less invasive and more abundant than intra-articular [12, 13]. Hence, use of subcutaneous adipose tissue ASCs may reduce morbidity and augment tissue resources for generation of intra-articular structures.

A recent study highlighted differences between ASCs from intra-articular and subcutaneous adipose tissues in and around the human knee [12]. Published reports of canine subcutaneous and infrapatellar ASCs suggest similarities between cells from the two harvest sites [10, 14], but a side by side in vitro characterization is necessary to anticipate the ability of each cell type to support complex tissue generation. This study was designed to determine in vitro expansion capabilities and plasticity of paired canine ASCs harvested from subcutaneous (SUB) and infrapatellar (IFP) adipose tissues before and after cryopreservation to test the hypothesis that IFP ASCs have the highest in vitro expansion rates, plasticity and MSC immunophenotypes that are sustained over multiple cell passages.

## Materials and Methods

### Ethics Statement

All animal procedures were approved by the Institutional Animal Care and Use Committee.

### Study Design

Subcutaneous and IFP adipose tissues were collected from six adult, mixed-breed dogs (3.5 ± 0.8 years of age, mean ± standard error of the mean (SEM)). Adipocyte and ASC ultrastructure were evaluated with transmission electron microscopy (TEM). Cell doublings (CDs) and doubling time (DT) were determined for fresh and cryopreserved ASC passages (fresh, P; cryopreserved, tP) 0–3 from both tissues of each dog. For P0, 1, 3 and tP1 and 3, CD29+, CD34+, CD44+, and CD90+ cell percentages were quantified. Fibroblastic (CFU-F), osteoblastic (CFU-Ob), and adipocytic (CFU-Ad) colony-forming unit frequency percentages and lineage-specific target gene mRNA expression levels were determined (peroxisome proliferator-activated receptor γ (PPAR-γ) and leptin - adipogenesis; osteoprotegerin (OPG) and collagen type 1α1 (Col1α1) - osteogenesis) after culture in lineage-specific induction or stromal media. Similarly, P3 and tP3 chondrogenic pellet alcian blue (proteoglycan) staining, CD29, CD34, CD44 and CD90 protein expression and target protein expression (PPAR-γ - adipogenesis; osteopontin (OPN) - osteogenesis; sex determining region Y-box 2 (SOX2) – progenitor) were also quantified following standard induction procedures. A seeding density of 5 × 10^3^ cells/cm^2^ and humidified culture conditions (37 °C, 5 % CO_2_) were used for all cell culture experiments. Materials were from Sigma-Aldrich, St. Louis, MO unless otherwise noted.

### Cell Isolation

Cells from SUB and IFP adipose tissues were isolated according to published procedures with minor modifications [11]. Briefly, tissue was rinsed with phosphate buffered saline (PBS, Hyclone, Logan, UT) and minced, followed by digestion in an equal volume of Dulbecco’s modified Eagle’s medium: F-12 (DMEM/F-12, Hyclone, Logan, UT), 0.1 % collagenase type I (Worthington Biochemical Corporation, Lakewood, NJ) and 1 % bovine serum albumin (BSA) for 90 min with agitation. The tissue suspension was then filtered (100 μm, BD Falcon, Bedford, MA). Subsequently, the digest was centrifuged (260 × g, 5 min), and the pellet was suspended in stromal medium (DMEM/F-12 medium, 10 % (v/v) fetal bovine serum (FBS, Hyclone), 1 % (v/v) antibiotic/antimycotic solution). An equal volume of red cell lysis buffer (0.16 mol/L NH_4_Cl, 0.01 mol/L KHCO_3_, 0.01 % ethylenediaminetetraacetic acid (EDTA)) was added, and the mixture was maintained at room temperature for 30 min. The solution was centrifuged, and the resulting stromal vascular fraction (SVF) pellet resuspended in stromal medium followed by culture in T75 flasks (CellStar, Greiner, NC). Medium was refreshed after 24 h to remove unattached cells, and then every 3 days [11]. Subsequent passages were performed at 70–80 % cell confluence. For purposes of this study, the primary cell isolate was the SVF and the first cell passage of primary cells was P0. Aliquots of each SVF were suspended in freezing medium (10 % DMEM/F-12, 10 % dimethyl sulfoxide (DMSO, Fisher Scientific, Fair Lawn, NJ), 80 % FBS) during the first cell passage and cryopreserved in liquid nitrogen for a minimum of 30 days. Cells were revitalized and seeded in T75 flasks as tP0 after the cryopreservation period.

### Adipocyte and ASC Ultrastructure (Adipocytes, P1, tP1) - Transmission Electron Microscopy

Harvested SUB and IFP adipose tissues, P1 and tP1 ASCs were rinsed with PBS and then fixed in 2 % paraformaldehyde and 1.25 % glutaraldehyde in 0.1 M sodium cacodylate (CAC) buffer (pH 7.4). Samples were rinsed in 0.1 M CAC buffer with 5 % sucrose and incubated with 1 % osmium tetroxide in 0.1 M CAC buffer. The tissues were dehydrated in a series of ethanol-distilled water solutions and embedded in Epon (Plano, Marburg, FRG). Ultrathin sections were evaluated with a transmission electron microscope (JEM-1011, JEOL, Japan).

### Cell Expansion (P0-3, tP1- 3)

Cells were cultured in 12-well plates (Thermal Fischer Scientific, Denmark) to calculate CD and DT after 2, 4 and 6 days of culture using standard formulae (CD = ln(Nf/Ni)/ln(2) and DT = CT/CD; Ni: initial cell number; Nf: final cell number; CT: culture time) [15]. Cell counts were performed with a hemocytometer. Day 2 and 4 cell numbers were used as the Ni for days 4 and 6, respectively.

### Multipotentiality (P0, 1, 3, tP1, 3) - Limiting Dilution Assays and Pellet Chondrogenesis

Limiting dilution assays were used to determine fibroblastic (CFU-F), adipocytic (CFU-Ad), and osteoblastic (CFU-Ob) colony-forming unit frequencies [3]. A total of 5000, 2500, 1250, 625, 312 or 156 cells were seeded in each well of one row in a 96-well plate for eight replicates of each cell number/well. Fibroblastic (CFU-F) colonies were fixed with 2 % formalin (Anapath, Mckinney, TX) and stained with 0.1 % toluidine blue after 7 days of culture in stromal medium. For CFU-Ob, cells were cultured in stromal medium for 7 days followed by culture in osteogenic medium (DMEM/F-12, 10 % FBS, 10 mmol/L β-glycerophosphate, 10 nmol/L dexamethasone, 50 μg/ml sodium 2-phosphate ascorbate) for 21 additional days. Cells were fixed in ice cold 70 % ethanol and then alkaline phosphatase (ALP) was stained with 5-bromo-4chloro-3indolyl phosphate/nitro blue tetrazolium (BCIP/NBT, 0.15 mg/ml BCIP, 0.30 mg/ml NBT). To determine CFU-Ad, cells were cultured in stromal medium for 7 days followed by culture in adipogenic medium (DMEM/F12, 3 % FBS, 1 % antibiotic/antimycotic solution, 33 μmol/L biotin, 17 μmol/L pantothenate, 1 μmol/L dexamethasone, 100 μmol/L indomethacin, 1 μmol/L insulin, 0.5 mmol/L isobutylmethylxanthine (IBMX), 5 μmol/L rosiglitazone (TZD, AK Scientific, Union City, CA)) for 21 additional days. Cells were fixed in 4 % formalin and stained with 0.3 % oil red O. Wells with 10 or more toluidine blue-stained, oil red O-stained, or BCIP/NBT-stained colonies were considered positive for fibroblastic, adipocytic or osteoblastic colonies, respectively. The ratio of negative to total wells per row was used to estimate the CFU frequencies according to Poisson’s ratio (F = e^-x^; F: ratio of negative to total wells; e: natural logarithm constant 2.71; x: CFU/well) [3, 11, 15]. CFU frequency was expressed as a percentage (1/CFU frequency × 100).

For chondrogenesis, 2.5 × 10^5^ cells were centrifuged (200×g, 5 min) to form a pellet after 7 days of culture in stromal medium. Pellets were cultured in stromal or chondrogenic medium (DMEM/F-12, 3 % FBS, 1 % antibiotic/antimycotic solution, 50 μg/ml ascorbate phosphate, 100 nmol/L dexamethasone, 40 μg/mL proline, 2 mmol/L sodium pyruvate (Invitrogen, Carlsbad, CA), 1 % insulin-transferrin-selenium (ITS, Invitrogen), 10 ng/mL recombinant human transforming growth factor-β3 (rTGF-β3, R&D systems, Minneapolis, MN)) for 21 days. They were then fixed with 10 % neutral buffered formalin, embedded in paraffin, sectioned (5 μm) and stained with 1 % alcian blue (Acros Organics, Belgium) and 3 % nuclear fast red.

### Immunophenotype - Flow Cytometry (P0, 1, 3, tP1, 3)

Cell aliquots (10^5^ cells) were suspended in 200 μl PBS containing 0.1 μl (200 μg/ml) of labeled or unlabeled antibody (CD34-PE, CD44-FITC, CD90-PE, CD29) specific for canine antigens or validated for canine cross reactivity for 30 min at room temperature (Table 1). Cells were then washed with PBS and fixed with 4 % neutral buffered formalin. For CD29, cells were incubated with labeled anti-immunoglobulin (IgG-FITC) for 30 min at room temperature, washed with PBS and then fixed with 4 % neutral buffer formalin. For the autoflourescence control, cells were not incubated with antibodies. Cell fluorescence was quantified by flow cytometry using a FACSCalibur flow cytometer and Cell Quest Pro software (BD Biosciences, San Jose, CA).

**Table 1:** Antibodies for Flow Cytometry and Immunocytochemistry

Antibody	Label	Marker expression	Manufacturer	Cat no.	Species	Target species	Diluent
CD29	N/A	β1 integrin	BD Biosciences	610468	Mouse	Human	PBS
CD34	PE	Hematopoietic progenitor (HSC)	BD Biosciences	559369	Mouse	Dog	PBS
CD44	FITC	Hyaluronic acid receptor	eBiosciences	115440	Mouse	Dog	PBS
CD90	PE	Thy-1, fibroblasts, MSC, HSC	eBiosciences	125900	Mouse	Dog	PBS
Goat anti-mouse IgG	FITC	Secondary antibody	Sigma-Aldrich	F9006	Goat	Mouse	PBS
β-actin	N/A	Cytoskeletal protein	Thermo Scientific	RB-9421-P0	Rabbit	Dog	TBS
Sox-2	N/A	Transcription factor	LifeSpan BioSciences	LS-B4562	Rabbit	Dog	5 % Skim milk/PBS
PPAR-γ	N/A	NHR/NR1 Thyroid homone-like	LifeSpan BioSciences	LS-B651	Rabbit	Dog	5 % BSA/TBS
OPN	N/A	Secreted Phosphoprotein 1	LifeSpan BioSciences	LS-B425	Rabbit	Dog	5 % BSA/TBS
CD29	N/A	β1 integrin	BD Biosciences	610468	Mouse	Human	TBS
CD44	N/A	Hyaluronic acid receptor	Monoclonal Antibody Center	DG-BOV2037	Mouse	Dog	TBS
Goat anti-rabbit IgG	HRP	Secondary antibody	Santa Cruz Biotechnology	sc-2004	Goat	Rabbit	TBS
Goat anti-mouse IgG	HRP	Secondary antibody	Santa Cruz Biotechnology	sc-2005	Goat	Mouse	TBS

### Gene Expression – RT-PCR (P0, 1, 3, tP1, 3)

Total RNA was isolated from cells (RNeasy Plus Mini Kit, Qiagen) and the concentration determined spectrophotometrically (NanoDrop ND-1000; NanoDrop Technologies, Montchanin, DE, USA). A QuantiTect Reverse Transcription Kit (Qiagen, GmbH, Germany) was used to generate cDNA. Target gene levels, glyceraldehyde 3-phosphate dehydrogenase (GAPDH), PPAR-γ, leptin, Col1α1 and OPG (Table 2) were quantified with real-time RT-PCR using SYBR Green (Qiagen) technology and an MJ Research Chromo 4 Detector (Bio-Rad Laboratories, Hercules, CA). The 2 ^–ΔΔCt^ values were determined relative to the reference gene GAPDH and target gene expression in cells cultured in stromal medium.

**Table 2:** Primer sequences

Lineage	Primer	Forward primer	Reverse primer	Accession no.
Housekeeping	GAPDH	TGGCAAAGTGGATATTGTCG	AGATGGACTTCCCGTTGATG	XM_003435649.2
Adipogenic	PPAR-γ	TTCTCCAGCATTTCCACTCC	AGGCTCCACTTTGATTGCAC	XM_005632014.1
	Leptin	TGTGTTGAAGCTGTGCCAAT	CCCTCTGTTTGGAGGAGACA	XM_005628342.1
Osteogenic	Col1α1	GGTGGTGGCTATGACTTTGG	CAGTTCTTGGCTGGGATGTT	XM_005628344.1
	OPG	TGTCTATACTGCAGGCCGGTG	TCAGGCAGAACTCAAGCTCCA	XM_003639448.2

### Protein Expression – Immunocytochemistry, Western Blot (P3, tP3)

#### Immunocytochemistry

Cells were cultured in stromal medium in 4-well chamber slides for 3 days and then fixed with 10 % neutral buffered formalin for 24 h. Antigen retrieval was performed with SDS/TBS antigen retrieval buffer (1 % SDS, 100 mM Tris, 138 mM NaCl, 27 mM KCl, pH = 7.4) for 5 min at room temperature. Cell preparations were blocked with goat serum (1:10 in TBS) overnight at 4 °C and then incubated with antibodies against CD34-PE (1:200), CD44-FITC (1:500) or CD90-PE (1:500) (Table 1) for 2 h at room temperature. Cells were washed with TBS buffer and nuclei were stained with DAPI (1 μg/ml). For CD29 labeling, cells were incubated with antibodies against CD29 (1:500) for 2 h at 37 °C followed by IgG-FITC (1:800) and DAPI nuclear stain. Photomicrographs were obtained for all labeled cells (Leica DM 4500b).

#### Western Blot

Total protein was extracted from cells cultured in stromal and induction medium using RIPA buffer (150 mM NaCl, 1.0 % Triton X-100 (Thermo Scientific, Rockford, IL), 0.5 % sodium deoxycholate, 0.1 % SDS, 50 mM Tris (pH = 8.0), protease inhibitor (Thermo Scientific)). Protein concentrations were determined with the Bicinchoninic Acid (BCA) Protein Assay Kit (Thermo Scientific). Protein aliquots (20 μg) were denatured for 10 min at 95 °C in an equal volume of 1× SDS loading buffer. Proteins were separated on a 12 % SDS-PAGE gel and transferred (300 mA, 90 min) to nitrocellulose membranes (Bio-Rad Laboratories, Hercules, CA). Membranes were blocked (4 % BSA, 0.05 % sodium azide) for 1 h at room temperature and then incubated with primary antibodies directed against OPN (1:300), PPAR-γ (1:500) or SOX2 (1:500) at 4 °C overnight (Table 1). Tris buffer saline with Tween-20 (TBST, 0.1 % Tween-20, 0.24 % Tris, 0.8 % NaCl) washes were followed by incubation with horseradish-peroxidase-conjugated (HRP) IgG (1:3000) at room temperature for 2 h. Labeled bands were imaged by exposure to light sensitive film (GE Healthcare, Chalfont St. Giles, Buckinghamshire, UK) following color development with Amersham ECL prime western blotting detection reagents (GE Healthcare). The relative density of target protein bands were determined normalized to β-actin standards (ImageJ v1.49p, National Institutes of Health, Bethesda, MD).

For evaluation of surface marker protein expression, total protein was extracted with NP-40 buffer (150 mM sodium chloride, 1.0 % Triton X-100, 50 mM Tris, pH = 8.0) from fresh and cryopreserved P3 cells cultured in stromal media. Protein concentration determination and western blot procedures were identical to above with the exception of incubation with primary antibodies directed against CD29 (1:200) and CD44 (1:2000) at room temperature for 1 h (Table 1) followed by IgG-HRP (1:2000).

### Statistical Analysis

Statistical analyses were performed with commercially available software (SAS 9.2, Institute, Cary, NC). Analysis of variance (ANOVA) models were used to evaluate cell surface marker percentages, CD, DT, CFU frequency percentages and gene expression among tissue sources within passages and among passages within tissue sources. Tukey’s post hoc tests were applied for multiple group comparisons (*p* < 0.05). Normality and homogeneity of variance were assessed with Shapiro-Wilk tests and residual plots.

## Results

### Adipocyte and ASC Ultrastructure (Adipocytes, P1, tP1) - Transmission Electron Microscopy

**Fig. 1:**
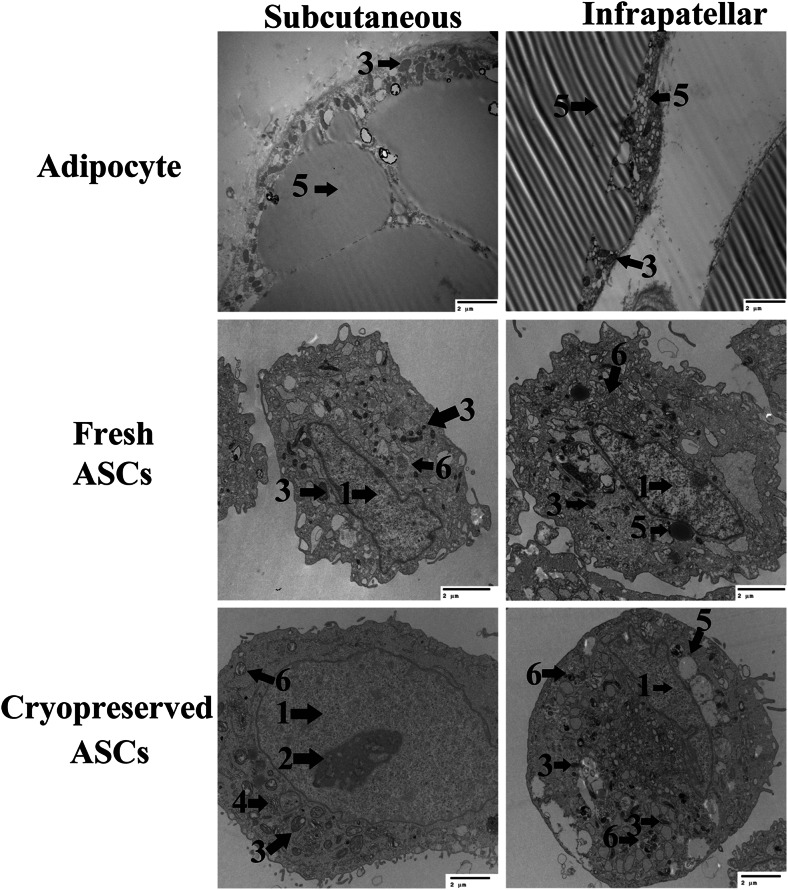
Transmission electron microscopy (TEM) image of adipocytes and adipose derived multipotent stromal cells (ASCs) from canine subcutaneous (SUB) and infrapatellar (IFP) adipose tissues. Legend: *1* – nucleus, *2* – euchromatin, *3* – mitochondria, *4* – Golgi apparatus, *5* – lipid droplet, *6* – lysosome. Scale bar = 2 μm

Adipocytes and ASCs had different morphologies from each other and between tissue sources. There were large, unilocular lipid vacuoles that peripherally displaced cytoplasm and organelles in adipocytes from both tissue sources. However, SUB adipocytes had numerous mitochondria concentrated near the cell membrane compared to mitochondria distributed throughout the cytoplasm in IFP adipocytes. Further, IFP adipocytes had abundant small lipid vacuoles (in addition to the central vacuole) among mitochondria whereas SUB had fewer (Fig. 1). Cultured ASCs were smaller than adipocytes, and they did not have central, large lipid vacuoles. Fresh IFP and SUB ASCs had mitochondria clustered around the nucleus and small, cytoplasmic lipid vacuoles, though IFP ASCs tended to have more vacuoles and fewer mitochondria. After cryopreservation, IFP ASCs had many small lipid vacuoles and few mitochondria in the cytoplasm compared to few lipid vacuoles in the cytoplasm and mitochondria clustered around the nucleus in SUB ASCs (Fig. 1). The perinuclear mitochondria apparent in the fresh IFP ASCs were not detectable in the same cells after cryopreservation. Additionally, the relative number of mitochondria in cryopreserved SUB ASCs appeared to be lower than in fresh ASCs based on subjective assessment.

### Cell Expansion (P0-3, tP1-3)

**Fig. 2:**
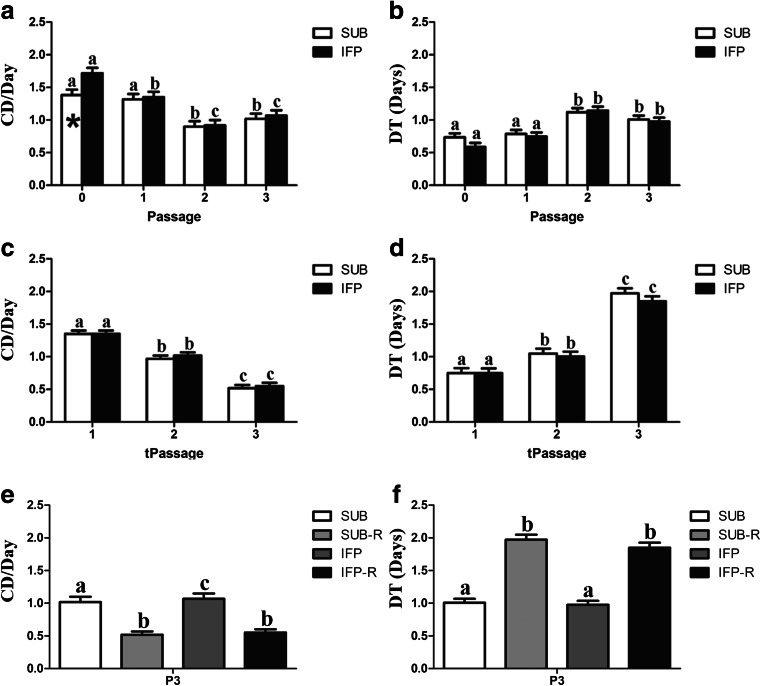
Cell doublings (CD) and doubling time (DT) (mean ± SEM) for fresh (**a**, **b**), revitalized (**c**, **d**) and both fresh and revitalized (-R) canine ASCs (**e**, **f**) from SUB and IFP adipose tissues. Columns with *asterisks* within passages are significantly different between cell tissue source (**a**), columns with distinct superscripts within cell tissue source (**a**–**d**), and columns with distinct superscripts within passage (**e**, **f**) are significantly different (*P* < .05)

The CDs of fresh and revitalized IFP and SUB ASCs decreased with passage, while the corresponding DTs increased (Fig. 2). Significant differences were less incremental in fresh versus cryopreserved cells with P0 and 1 tending to expand more quickly than P2 and 3. The CDs were significantly lower for P0 SUB ASCs versus IFP ASCs (Fig. 2a). The CDs were significantly lower (Fig. 2e) and the DT significantly higher (Fig. 2f) for cryopreserved P3 versus fresh ASCs from the same tissue source.

### Multipotentiality (P0, 1, 3, tP1, 3) – Limiting Dilution Assays and Pellet Chondrogenesis

**Fig. 3:**
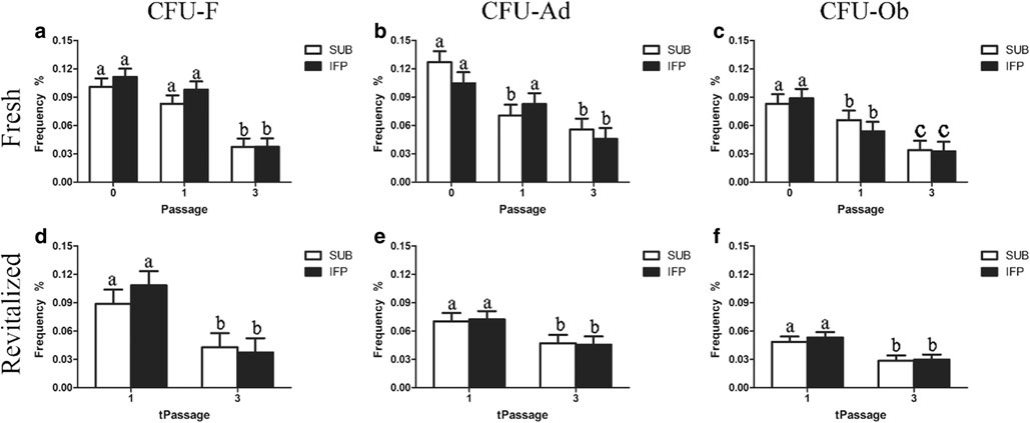
Colony forming unit (CFU) frequencies (mean ± SEM) for fresh (**a**, **b**, **c**) and revitalized (**d**, **e**, **f**) canine ASCs from subcutaneous (SUB) or infrapatellar (IFP) adipose tissue after culture in stromal (CFU-F), osteogenic (CFU-Ob), or adipogenic (CFU-Ad) medium. Columns with different superscripts within cell source are significantly different among passages (*P* < .05)

**Fig. 4:**
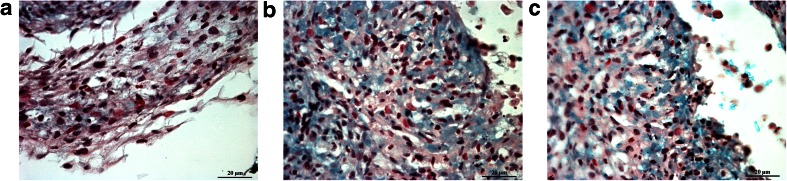
Light photomicrographs of fresh P3 canine ASCs from SUB (**a**, **b**) and IFP (**c**) after pellet culture in stromal (**a**) or chondrogenic (**b**, **c**) medium. Alcian blue staining of proteoglycans with nuclear fast red counter stain. 63× magnification. Scale bar = 20 μm

Based in histochemical staining, fresh and revitalized IFP and SUB ASCs showed robust adipogenesis and osteogenesis for all passages evaluated. The CFU frequency percentage indicates the percentage of cells capable of forming a colony of fibroblastic (CFU-F), osteoblastic (CFU-Ob) or adipogenic (CFU-Ad) lineage. Before and after cryopreservation, SUB and IFP ASCs showed similar CFU frequency percentages, and, though values tended decrease after cryopreservation, they were not significantly different among fresh and cryopreserved cells from the same tissues within passages (Fig. 3). In fresh cells, the frequencies decreased with passage and were significantly lower in P3 versus P0. For CFU-F, P0 and 1 were significantly higher than P3, and for CFU-Ob, P0 was significantly higher than P1 which was significantly higher than P3. The only difference in behavior between cell sources was that P1 SUB ASC CFU-Ad was significantly lower than P0 while IFP ASC CFU-Ad was significantly higher in P0 and 1 than P3 in fresh cells. Fresh SUB and IFP ASCs displayed characteristic chondrogenic differentiation including glycosaminoglycan (GAG) deposition and tissue organization, both of which were absent in the control group (Fig. 4).

### Immunophenotype -Flow Cytometry (P0, 1, 3, tP1, 3)

**Fig. 5:**
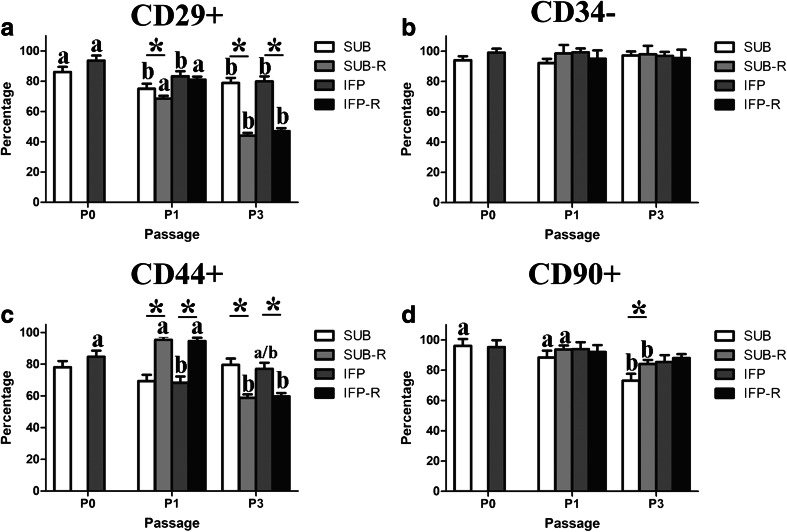
Percentages (mean ± SEM) of CD29+ (**a**), CD34- (**b**), CD44+ (**c**) and CD90+ (**d**) fresh and revitalized (−R) ASCs from canine SUB and IFP adipose. Columns connected by lines with asterisks are significantly different within passage and columns with distinct superscripts within cell tissue source and treatment (fresh or cryopreserved) are significantly different among passages (*P* < .05)

The majority of fresh cells from both adipose tissue depots were CD29+, CD44+, CD90+ and CD34- for all passages evaluated (Fig. 5). The percentage of CD29+ ASCs was significantly higher in P0 compared to 1 and 3 for fresh SUB and IFP ASCs (Fig. 5a). There were significantly higher percentages of CD29+ and CD44+ ASCs in revitalized P1 versus P3 for both tissue sources (Fig. 5a, c). Fresh P1 SUB and P3 SUB and IFP ASCs had significantly higher percentages of CD29+ cells than the same passages after cryopreservation (Fig. 5a). There were significantly higher percentages in of CD44+ P1 ASCs after cryopreservation compared to fresh cells within tissue source (Fig. 5c). However, cryopreserved P3 ASCs had significantly lower percentages of CD44+ than fresh. The percentage CD90+ cells was significantly lower in fresh P3 SUB ASCs compared to cryopreserved P3 SUB ASCs (Fig. 5d).

### Gene Expression - RT-PCR (P0, 1, 3 tP1, tP3)

**Fig. 6:**
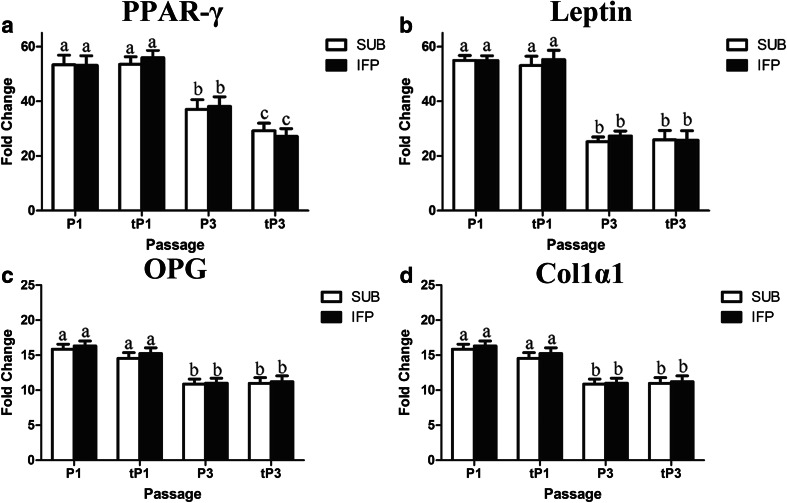
Fold change (mean ± SEM) of adipogenic (**a**, **b**) and osteogenic (**c**, **d**) lineage target genes in fresh and revitalized (t) canine ASCs from subcutaneous (SUB) and infrapatellar (IFP) adipose tissue cultured in induction medium. Values are relative to the reference gene GAPDH and to cells cultured in stromal medium. PPAR-γ = Peroxisome proliferator-activated receptor γ, *OPG* = Osteoprotegerin, *Col 1α1* = Collagen Iα1. Columns with different superscripts within cell tissue source are significantly different (*P* < .05)

The stability of the reference gene, GAPDH, was confirmed based on a mean CT value of 18.18 ± 0.22 for all samples. With increasing passage, adipocytic and osteoblastic target gene expression decreased significantly in both fresh and cryopreserved cells (Fig. 6). Both P1 and tP1 SUB and IFP ASCs had significantly higher levels of PPAR-γ and leptin after adipogenic induction (Fig. 6a, b) as well as OPG and Col 1α1 after osteogenic induction compared to P3 and tP3 (Fig. 6c, d). Additionally, PPAR-γ expression was significantly in lower in P3 ASCs following induction after cryopreservation in cells from both tissue sources.

### Protein Expression – Immunocytochemistry, Western Blot (P3, tP3)

#### Immunocytochemistry

**Fig. 7:**
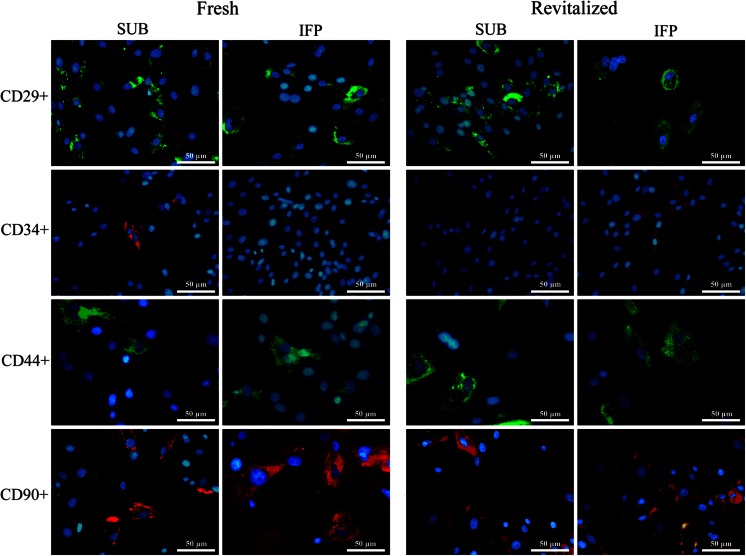
Fluorescent photomicrographs of SUB and IFP fresh and revitalized canine P3 ASCs from subcutaneous (SUB) and infrapatellar (IFP) adipose tissue labeled with cell surface markers indicated. 40× magnification. Scale bar = 50 μm

Cell surface marker expression (CD29+, CD44+, CD90+, CD34−) was confirmed with immunocytochemistry in all ASCs before and after cryopreservation (Fig. 7).

#### Western Blot

**Fig. 8:**
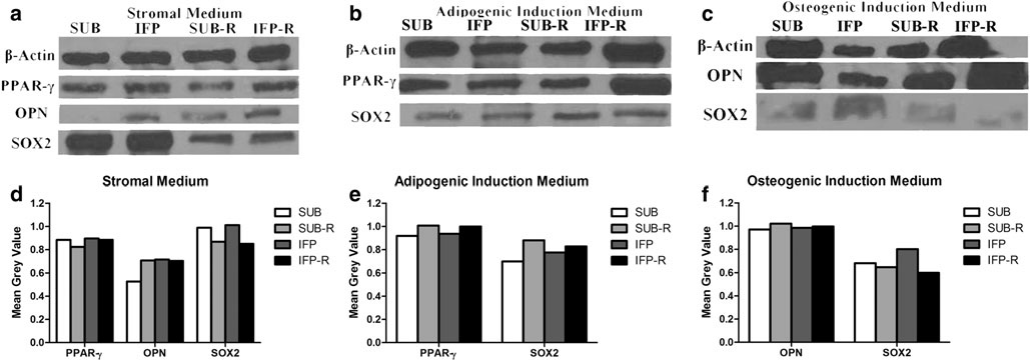
Western blot image of proteins from fresh and revitalized (−R) canine P3 ASCs from subcutaneous (SUB) and infrapatellar (IFP) adipose tissue after culture in stromal (**a**), adipogenic (**b**) or osteogenic (**c**) medium with corresponding graphs indicating relative density (**d–f**). *PPAR-γ* = Peroxisome proliferator-activated receptor-γ, *SOX2* = Sex determining region Y box-2, *OPN* = Osteopontin

**Fig. 9:**
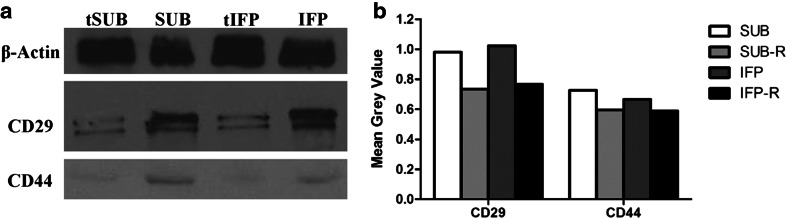
Western blot image (**a**) and corresponding relative density graph (**b**) of fresh and revitalized (t) P3 ASCs following culture in stromal medium

The SOX2 protein expression was lower in cryopreserved (-R) ASCs from both tissues cultured in stromal medium (Fig. 8a, d). Additionally PPAR-γ and OPN protein expression increased while SOX2 expression decreased in fresh and revitalized P3 ASCs cultured in adipogenic and osteogenic induction medium, respectively (Fig. 8b–f). The singular exception was revitalized P3 SUB ASCs in which SOX2 expression was essentially the same in cells cultured in adipogenic and stromal medium. Cryopreserved P3 SUB and IFP ASCs had lower CD29 and CD44 protein expression compared to fresh P3 ASCs (Fig. 9).

## Discussion

Paired comparisons of fresh and revitalized ASCs from canine SUB and IFP adipose tissue in this study showed comparable in vitro expansion and multipotentiality up to passage 3. Fresh and cryopreserved canine ASC expansion metrics, CD and DT, and CFU frequency percentages had similar values and cell passage changes to previous reports using the same [3, 11]. However, detectable effects of cryopreservation on ultrastructure, immunophenotype, and both surface marker and transcription factor expression indicates that cryopreserved canine ASCs may not be directly interchangeable with fresh. In spite of the potential impact of cryopreservation on ASC potential, study results suggest that ASCs from either tissue source are promising candidates for future studies to optimize cell isolation, cryopreservation, tissue regeneration strategies and therapeutic applications.

The ultrastructures observed in this study were similar to those reported for other species [16, 17] including abundant cytoplasmic mitochondria [16] and many small lipid vacuoles [17] in brown and white adipocytes, respectively. The characteristics are consistent with lipid energy storage [8, 18] and the role of the inner mitochondrial membrane in thermogenesis [19, 20]. Mature adipocytes are post-mitotic, so cell precursors rapidly differentiate in response to energy demands [21, 22]. As such, immature cells at many stages of differentiation assume characteristics of the mature cells as observed in the ASCs in study. This is also consistent with the current knowledge that white and brown adipocytes are derived from distinct precursor populations [23–25].

Fewer mitochondria surrounding the nucleus after cryopreservation may indicate greater maturity in revitalized cells. Mitochondrial localization is a characteristic of mammalian cleavage-stage embryos that is strongly indicative of stem cell competence, and alterations may be associated with diminished pluripotency [26, 27]. The percentage of cells with perinuclear mitochondria decreased while that of cells with lipid droplets increased with cell passage in an adult primate adipose derived stem cell line [26]. Reduced perinuclear mitochondria were associated with lower oxygen consumption and higher adenosine triphosphate, both consistent with lower metabolic energy requirements of differentiated cells. The decrease in mitochondria after cryopreservation in this study was a subjective assessment, so the magnitude of the change could not be quantified or directly compared between ASC tissue sources. A consistent finding was that the distribution of mitochondria was similar within cells from specific tissue sources and the relative prevalence was reduced following cryopreservation.

Current knowledge suggests that loss of mitochondria may be a result of organelle disruption and/or cell selection by the cryopreservation process [28, 29]. The potential for cell selection is further supported by increased numbers of lipid vacuoles post-cryopreservation. The cryoprotectant used in this study, DMSO, is a standard component of cryopreservation media for cell protein and membrane stabilization [29, 30] and to prevent intracellular ice formation [29]. At non-toxic DMSO levels, and despite a slow freezing process, intracellular ice formation is not fully prevented [31–33]. Fewer mitochondria and more abundant lipid vacuoles are associated with greater cell maturity as indicated above [26, 27]. Based on the these findings, it is possible that the higher lipid content of more mature cells within the heterogeneous cell isolate confers a higher transition temperature during freezing [34]. Together with the DMSO, the higher lipid content may protect against ice formation and thereby select for greater cell maturity. Further studies are required for confirmation of this finding.

Additional distinctions between fresh and cryopreserved ASCs were decreases in cell percentages expressing CD29 and CD44, multipotent stromal cell phenotypic markers that are stably expressed in ASCs [2]. Exceptions included variable CD90 expression and an initial increase in the number of CD44+ cells in P1 cryopreserved cells. Previous studies identified increased CD90 expression in revitalized cells versus fresh MSCs from human umbilical cord and rat adipose and bone marrow tissues [35, 36]. It is generally accepted that due to the presence of CD90 in the endothelial population, changes in expression are best interpreted in combination with the expression of other surface markers [2]. The initial increase in CD44 expression in revitalized cells that was not paralleled in CD29 expression could be due to an increase in CD44 expression by cells that do not express CD29, potentially due to high protein concentrations in FBS. The principle ligand of CD44 is hyaluronic acid (HA) [37]. Murine cumulus cell-oocyte complexes retain HA in the extracellular matrix and expand in the presence of FBS [38]. In contrast, HA is released into the culture medium in the absence of FBS, the cells dissociate and attach to culture ware as individual cells [38]. Hence, high concentrations of FBS in the freezing medium may increase local HA concentrations and stimulate upregulation of the receptor in cells in the passage immediately post revitalization.

In general, there were fewer cells expressing CD29 and CD44 after cryopreservation within passages, with the exceptions discussed above, and passage effect tended to be more profound in cryopreserved cells. Lower CD29 and CD44 expression was confirmed by western blot. Upregulation of apoptotic genes and increased apoptosis is associated with cryoprotectants and the cryopreservation process itself [30, 39]. Additionally, DMSO concentrations of 10 % or higher reduce the number of porcine ASC fibroblastic colony forming units and increase cell apoptotic gene expression after cryopreservation [30]. A previous study confirmed that the expression of adhesion molecule CD62L [40] was lost while CD57, an apoptosis protein [41], increased after cryopreservation in CD4+ and CD8+ T cells [39]. Based on this established information, decreases in CD44 and CD29 post cryopreservation suggests a similar and important effect on canine SUB and IFP ASCs that should be considered for cell therapy and tissue regeneration strategies.

Decreases in SOX2 protein expression and PPAR-γ gene expression observed in this study further suggests that cryopreservation may impact ASC plasticity. Both are early transcription factors, SOX2 is associated with the multipotent ASC phenotype [42, 43] and PPAR-γ is a nuclear receptor that controls energy metabolism and cell differentiation that is highly expressed in ASCs [44, 45]. It further controls transcription of genes for radical oxygen species detoxification enzymes [46]. Recently, mitochondria were shown to be central to oligodendrocyte differentiation and major targets of PPAR-γ agonist protection against inflammatory cytokine damage [44]. The fact that PPAR-γ protein expression was not significantly lower in P3 ASCs after cryopreservation is likely because gene and protein expression is not necessarily parallel, and changes in gene expression typically precede alterations in protein levels. Lower expression of both SOX2 and PPAR- γ in addition to the reduced presence of cellular mitochondria may indicate greater cell vulnerability to inflammatory byproducts in addition to decreased differentiation capacity.

As a whole, these in vitro study results indicate that there are few differences between canine SUB and IFP ASCs before and after cryopreservation. The impact of cryopreservation on the cells is strongly supported at the genetic, protein and ultrastructural levels and appears to be consistent with reduced expansion capacity and plasticity associated with greater cell maturity. Cryopreservation is vital to storage and transport of progenitor cells [47–49]. It is especially important given the growing recognition of the importance of cell phenotype for specific therapeutic and tissue regeneration purposes [50–52]. Continued efforts to minimize the impact of cryopreservation on ASC properties as well mechanisms to clearly delineate and anticipate the effects will continue to improve cell-based therapies and tissue regeneration.
